# Association between frailty index and erectile dysfunction: A cross-sectional study using NHANES 2001 to 2004 data

**DOI:** 10.1097/MD.0000000000048464

**Published:** 2026-05-08

**Authors:** Wei Wu, Peihe Liang

**Affiliations:** a Department of Urology, The Second Affiliated Hospital of Chongqing Medical University, Chongqing, China.

**Keywords:** diagnostic performance, erectile dysfunction, frailty index, NHANES

## Abstract

The objective of this study was to examine the association between the frailty index (FI) and erectile dysfunction (ED) in the general US population. We utilized data from the National Health and Nutrition Examination Survey 2001 to 2004 on erectile function and the FI. ED was assessed via a self-reported questionnaire. The FI was calculated using a 49-item model and analyzed both as a continuous and a categorical variable. Participants were stratified into 3 groups: non-frail (FI ≤ 0.15), pre-frail (0.15 < FI ≤ 0.25), and frail (FI > 0.25). The association between the FI and ED was evaluated using weighted logistic regression across 3 models: model 1 (unadjusted), model 2 (adjusted for age, race, poverty-income ratio, marital status, and education level), and model 3 (further adjusted for additional potential covariates from model 2). Nonlinear associations were examined using restricted cubic spline, supplemented by subgroup analyses and interaction tests. Finally, receiver operating characteristic curve analysis was performed to assess diagnostic performance. This study included a total of 1372 participants, among whom 674 were diagnosed with ED. After adjusting for all covariates, a significant positive association was observed between the FI and ED (odds ratio [OR]: 1.09, 95% confidence interval [CI]: 1.06–1.19, *P* < .001). Using the non-frail group as reference, similar associations were found for both the pre-frail group (OR: 1.09, 95% CI: 1.03–1.16, *P* = .011; *P* for trend < .001) and frail group (OR: 1.22, 95% CI: 1.13–1.30, *P* < .001; P for trend < .001). Restricted cubic spline and smooth curve fitting demonstrated a linear relationship between FI and ED risk. Stratified analyses and interaction tests further confirmed the positive association between FI and ED prevalence. Receiver operating characteristic curve analysis (area under the curve = 0.662, 95% CI: 0.633–0.690) indicated that FI had moderate predictive accuracy for ED diagnosis. This study demonstrates a significant positive association between the FI and ED, with FI showing substantial predictive accuracy for ED. These findings suggest FI may serve as a valuable biomarker for ED prevention and clinical diagnosis.

## 1. Introduction

Erectile dysfunction (ED) is a common male sexual dysfunction, defined as the persistent inability to achieve or maintain a penile erection sufficient for satisfactory sexual function. Statistics show that the prevalence rate of ED among men nationwide in the United States reaches 24.2%, and 17.9% of young men under 24 years old meet the diagnostic criteria for ED, indicating a trend of ED affecting younger individuals.^[1]^ It is estimated that the number of people suffering from ED worldwide will increase from 152 million in 1997 to 322 million in 2025.^[2]^ ED not only deteriorates the quality of men’s sexual life and affects fertility but also often predicts potential cardiovascular diseases (CVDs) or other serious health conditions.^[3,4]^ The occurrence of ED is brought about by the combined action of multiple whole-body systems and involves multiple etiological factors such as vascular, neurogenic, endocrine, and psychosocial aspects.^[5–7]^ Existing studies have proven the correlation between ED and various medical and lifestyle factors, including diabetes, hypertension, depression, obesity, sleep disorders, CVDs, drug factors, and endocrine diseases.^[8,9]^

Frailty is a clinical syndrome characterized by multisystem functional decline. We quantified the degree of frailty using the frailty index (FI) proposed by Rockwood et al, which integrates multidimensional variables such as cognitive function, dependence in daily activities, depressive symptoms, comorbidities, medical utilization, physical performance, and laboratory indicators (CVDs, diabetes, physical decline, depressive mood, etc).^[10,11]^ The associations between individual factors covered by the FI (such as CVDs, depression, and physical decline) and ED have been partially confirmed. However, there is still a lack of systematic research on the association between the FI as an overall assessment tool and ED.

From a pathophysiological perspective, there may be a profound link between frailty and ED. The 2 conditions share multiple pathways, such as vascular endothelial dysfunction, chronic low-grade inflammation, hormonal changes, and psychosocial stress.^[12–15]^ These common biological underpinnings suggest that frailty is not only a composite manifestation of multiple chronic diseases but may itself directly or indirectly contribute to the development and progression of ED through the aforementioned mechanisms. Therefore, investigating the association between frailty – as a holistic indicator of health deficit – and ED has a solid theoretical foundation. However, existing studies have predominantly focused on the relationship between individual diseases or symptoms (e.g., CVD, depression) and ED, while evidence systematically examining their epidemiological association using comprehensive frailty assessment tools, such as the FI, remains scarce.

To address this research gap, the present study was conducted using data from the National Health and Nutrition Examination Survey (NHANES) database, aiming to explore the relationship between the FI and ED.

## 2. Methods

### 2.1. Study design and participants

This study relied on secondary analysis of existing data from the NHANES database rather than collecting primary data. NHANES is a comprehensive research project designed to assess the health and nutritional status of the US population. It collects data from representative samples through a stratified, multi-stage probability cluster sampling method, covering data modules such as demographics, dietary habits, physical examinations, laboratory tests, and questionnaires. The 2001–2004 cycle of NHANES was used for this study because it was the sole cycle that included data on ED.^[16,17]^ It must be noted that the data are now approximately 2 decades old, and their dated nature may limit the direct applicability of the findings to contemporary populations. Nevertheless, the primary aim of this research is to elucidate the fundamental epidemiological relationship between frailty and ED – an association grounded in shared and relatively stable pathophysiological mechanisms, including vascular dysfunction, chronic inflammation, and hormonal alterations. These biological underpinnings are unlikely to change substantially over time. Consequently, despite the temporal lag in the data, the direction and magnitude of the association demonstrated herein remain robust and valid. The NHANES protocol has been reviewed and approved by the Research Ethics Review Committee of the National Center for Health Statistics, ensuring compliance with ethical guidelines and protection of participants’ rights. All detailed NHANES study designs and data are publicly available at https://www.cdc.gov/nchs/nhanes/.

**Figure 1.**
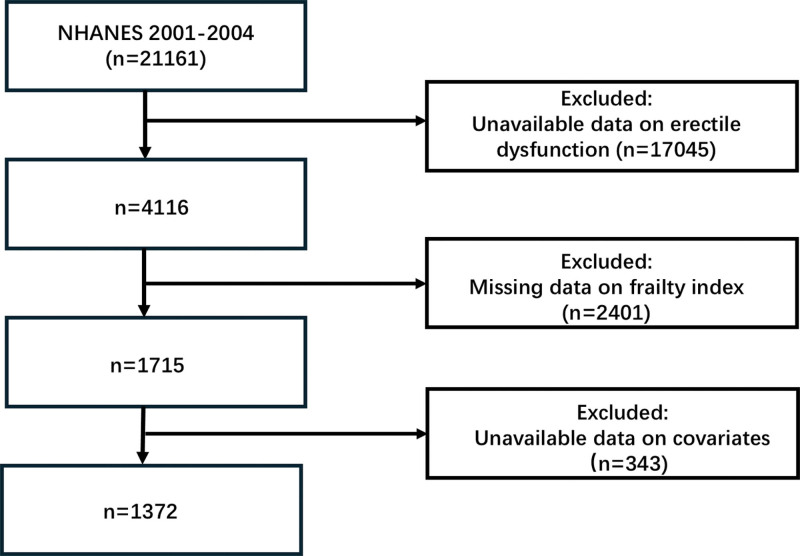
Flow chart of participant selection process of NHANES 2001 to 2004. NHANES = National Health and Nutrition Examination Survey.

In our cross-sectional study utilizing NHANES data from the 2001 to 2004 cycles, we initially included 21,161 participants. However, certain participants were excluded based on the following criteria: missing data related to ED (n = 17,045); missing data for components of the FI (n = 2401); and missing data for relevant covariates (n = 343). The final analytical sample consisted of 1372 participants (Fig. 1).

### 2.2. Data collection

#### 2.2.1. Assessment of ED

The assessment of ED was conducted in a private room at the mobile examination center using an audio computer-assisted self-interview format. During this interview, participants were asked to describe their ability to achieve and maintain an erection sufficient for satisfactory sexual activity. Response options included “always or almost always able,” “usually able,” “sometimes able,” and “never able.” Participants who selected “always or almost always able” or “usually able” were classified as non-ED, while those who chose “sometimes able” or “never able” were classified as having ED.^[18,19]^

#### 2.2.2. Measurement of FI

The FI used in this research is a composite measure constructed post hoc by the authors, strictly following the classic Rockwood model, utilizing existing variables within the survey rather than being a directly measured variable in the original dataset. The model consists of 49 health deficits across different systems, including cognition (memory loss and cognitive challenges), dependence (difficulty in performing daily activities), depressive symptoms, comorbidities (various chronic diseases), hospital utilization and general health, physical performance, physical assessments (grip strength and body mass index [BMI]), and laboratory data (including blood cell counts and blood glucose levels). Different responses to each health deficit correspond to different scores (see Table S1, which illustrates the variables composing the FI and their respective scores).^[10]^ The FI was calculated using the formula, FI = total score of measured deficits/number of measured deficits, resulting in FI data ranging from 0 to 1. For analytical purposes, we converted the FI into a categorical variable based on cutoff values established in previous studies: non-frail group (FI ≤ 0.15), pre-frail group (0.15 < FI ≤ 0.25), and frail group (FI > 0.25).^[11]^

#### 2.2.3. Covariates

Covariates, other than ED and FI, were selected based on their potential influence on ED and included age, race, education level, poverty-income ratio (PIR), marital status, BMI, smoking behavior, alcohol intake, moderate activity, hypercholesterolemia, diabetes, CVD, and hypertension. Race was categorized into 4 groups: Mexican American, non-Hispanic White, non-Hispanic Black, and Other Race. Education level was divided into 3 groups: <high school, high school, and >high school. PIR was classified as <2, 2 to 4, and >4. Marital status was dichotomized into living with a partner and living alone. BMI was categorized as <25, 25 to 30, and ≥30. Smoking and alcohol intake statuses were each classified into 3 groups: never, current, and former. Moderate activity was assessed via questionnaire. Hypercholesterolemia was diagnosed based on self-reported diagnosis, specific cholesterol levels, or use of lipid-lowering medications.^[20]^ Diabetes was diagnosed based on self-reported diagnosis, use of antidiabetic medications, glycated hemoglobin ≥ 6.5%, or fasting blood glucose ≥ 7.0 mmol/L.^[21]^ Hypertension was defined as self-reported diagnosis, use of antihypertensive medications, systolic blood pressure ≥ 140 mm Hg, or diastolic blood pressure ≥ 90 mm Hg.^[22]^ CVD was defined as a previous diagnosis of congestive heart failure, coronary heart disease, angina, heart attack, or stroke.^[23]^

### 2.3. Statistical analysis

The study population was divided into 2 groups based on the presence or absence of ED. Continuous variables were expressed as mean and standard error, while categorical variables were presented as frequency and proportion. To describe the characteristics of the study population, weighted chi-square tests were used to compare categorical variables between the ED and non-ED groups, and weighted *t* tests were applied for continuous variables.

The relationship between the FI and the prevalence of ED was investigated using a weighted logistic regression model, with 3 logistic regression models established: model 1 (unadjusted); model 2: adjusted for age, race, PIR, marital status, and education level; and model 3: adjusted for the factors in model 2 plus the remaining potential covariates. The FI was analyzed both as a continuous variable (per 0.1 increment) and a categorical variable.

To explore the nonlinear relationship between the FI and ED, restricted cubic spline (RCS) with smooth curve fitting was employed. Weighted logistic regression was used to conduct subgroup analyses and evaluate interaction effects across different demographic and clinical strata. Finally, receiver operating characteristic (ROC) curve analysis was performed to assess the predictive efficacy of the FI for ED. All statistical analyses were performed using R software (version 4.4.3; R Foundation for Statistical Computing, Vienna, Austria). Differences were considered statistically significant when *P* < .05.

## 3. Results

### 3.1. Participant characteristics

Among the 1372 participants in this study, 674 were diagnosed with ED, and the remaining 698 non-ED participants served as controls. Table 1 shows that the mean value of the FI in the ED group was significantly higher than that in the non-ED group (0.23 vs 0.16, *P* < .001). When the FI was treated as a categorical variable, the proportion in the ED group increased with the elevation of FI, while the proportion in the non-ED group decreased with the elevation of FI, and the difference was statistically significant (*P* < .001). Statistically significant differences were observed between the ED and non-ED populations in terms of age, race, PIR, education level, smoking status, CVD, hypertension, diabetes, and moderate activity.

**Table 1:** Demographic and clinical characteristics of participants, weighted.

Characteristic	Erectile dysfunction			*P* value
	n = 1372	Non-ED (n = 698)	ED (n = 674)	
FI, mean (SE)	0.20 (0.12)	0.16 (0.11)	0.23 (0.13)	<.001
FI group (%)				
Non-frail (FI ≤ 0.15)	578 (42.1%)	379 (54.3%)	199 (29.5%)	<.001
Pre-frail (0.15 < FI ≤ 0.25)	425 (31.0%)	192 (27.5%)	233 (34.6%)	
Frail (FI > 0.25)	369 (26.9%)	127 (18.2%)	242 (35.9%)	
Age, mean (SE) (yr)	63.7 (14.8)	57.6 (15.8)	70.0 (10.6)	<.001
Race (%)				
Mexican American	221 (16.1%)	98 (14.0%)	123 (18.2%)	.044
Non-Hispanic White	870 (63.4%)	441 (63.2%)	429 (63.6%)	
Non-Hispanic Black	217 (15.8%)	120 (17.2%)	97 (14.4%)	
Other Race	64 (4.66%)	39 (5.59%)	25 (3.71%)	
PIR (%)				
<2	543 (39.6%)	258 (37.0%)	285 (42.3%)	.002
2.0 ≤ PIR < 4.0	430 (31.3%)	207 (29.7%)	223 (33.1%)	
≥4.0	399 (29.1%)	233 (33.4%)	166 (24.6%)	
Education (%)				
<High school	438 (31.9%)	188 (26.9%)	250 (37.1%)	<.001
High school	313 (22.8%)	174 (24.9%)	139 (20.6%)	
>High school	621 (45.3%)	336 (48.1%)	285 (42.3%)	
Marital status (%)				
Cohabitation	1037 (75.6%)	512 (73.4%)	525 (77.9%)	.058
Solitude	335 (24.4%)	186 (26.6%)	149 (22.1%)	
BMI (%)				
Normal (<25)	369 (26.9%)	183 (26.2%)	186 (27.6%)	.349
Overweight (25–<30)	609 (44.4%)	323 (46.3%)	286 (42.4%)	
Obese (>30)	394 (28.7%)	192 (27.5%)	202 (30.0%)	
Smoking status (%)				
Never	439 (32.0%)	243 (34.8%)	196 (29.1%)	<.001
Current	297 (21.6%)	185 (26.5%)	112 (16.6%)	
Former	636 (46.4%)	270 (38.7%)	366 (54.3%)	
CVD				
Yes	299 (21.8%)	109 (15.6%)	190 (28.2%)	<.001
No	1073 (78.2%)	589 (84.4%)	484 (71.8%)	
Hypercholesterolemia (%)				
No	733 (53.4%)	389 (55.7%)	344 (51.0%)	.091
Yes	639 (46.6%)	309 (44.3%)	330 (49.0%)	
Hypertension (%)				
No	569 (41.5%)	343 (49.1%)	226 (33.5%)	<.001
Yes	803 (58.5%)	355 (50.9%)	448 (66.5%)	
Diabetes (%)				
No	1084 (79.0%)	596 (85.4%)	488 (72.4%)	<.001
Yes	288 (21.0%)	102 (14.6%)	186 (27.6%)	
Alcohol intake (%)				
Current	1110 (80.9%)	573 (82.1%)	537 (79.7%)	.522
Former	157 (11.4%)	75 (10.7%)	82 (12.2%)	
Never	105 (7.65%)	50 (7.16%)	55 (8.16%)	
Moderate activity (%)				
No	1082 (78.9%)	517 (74.1%)	565 (83.8%)	<.001
Yes	290 (21.1%)	181 (25.9%)	109 (16.2%)	

For continuous variables: presented as means with SEs; for categorical variables: displayed as counts (n) and percentages (%).

BMI = body mass index, CVD = cardiovascular disease, FI = frailty index, PIR = poverty-income ratio, SE = standard error.

### 3.2. The association between FI and ED

Table 2 presents the relationship between FI levels and ED. In model 1, for every 0.1 increment in the FI, the risk of ED significantly increased (odds ratio [OR]: 1.12, 95% confidence interval [CI]: 1.10–1.14, *P* < .001). Compared with the non-frail group (FI ≤ 0.15), participants in the pre-frail group (OR: 1.19, 95% CI: 1.12–1.26, *P* < .001) and the frail group (OR: 1.36, 95% CI: 1.27–1.45, *P* < .001) both showed a significantly higher risk of ED, with the risk of ED in the frail group being higher than that in the pre-frail group. The above associations persisted after sequential adjustments. In model 2 (adjusted for demographic factors), similar relationships were observed for the continuous FI (OR: 1.09, 95% CI: 1.07–1.11, *P* < .001), the pre-frail group versus the non-frail group (OR: 1.10, 95% CI: 1.05–1.16, *P* < .001), and the frail group versus the non-frail group (OR: 1.24, 95% CI: 1.17–1.32, *P* < .001). In the final model, after adjusting for all covariates, including demographics, lifestyle factors, and comorbidities, consistent results were shown for the continuous FI (OR: 1.09, 95% CI: 1.06–1.12, *P* < .001), the pre-frail group versus the non-frail group (OR: 1.09, 95% CI: 1.03–1.16, *P* < .001), and the frail group versus the non-frail group (OR: 1.22, 95% CI: 1.13–1.30, *P* < .001). In all 3 models, the trend test (*P* for trend) indicated that with the progression of FI groups (non-frail → pre-frail → frail), the risk of ED showed a statistically significant increasing trend, meaning there was a dose–response relationship between the FI and the risk of ED.

**Table 2:** Association between frailty index and erectile dysfunction across different regression models.

Characteristic	Model 1	Model 2	Model 3
OR (95% CI), *P* value	OR (95% CI), *P* value	OR (95% CI), *P* value
Frailty index (per 0.1 change)	1.12 (1.10–1.14), <.001	1.09 (1.07–1.11), <.001	1.09 (1.06–1.12), <.001
Frailty index group			
Non-frail (FI ≤ 0.15)			
Pre-frail (0.15 < FI ≤ 0.25)	1.19 (1.12–1.26), <.001	1.10 (1.05–1.16), <.001	1.09 (1.03–1.16), .011
Frail (FI > 0.25)	1.36 (1.27–1.45), <.001	1.24 (1.17–1.32), <.001	1.22 (1.13–1.30), <.001
*P* for trend	<.001	<.001	<.001

Model 1: No covariates adjusted.

Model 2: Adjusted for age, race, marital status, education level, and poverty-income ratio.

Model 3: Adjusted for model 2 + body mass index, alcohol intaking, cigarette smoking, physical activity status, diabetes, hypertension, and cardiovascular disease.

95% CI = 95% confidence interval, FI = frailty index, OR = odds ratio.

**Figure 2.**
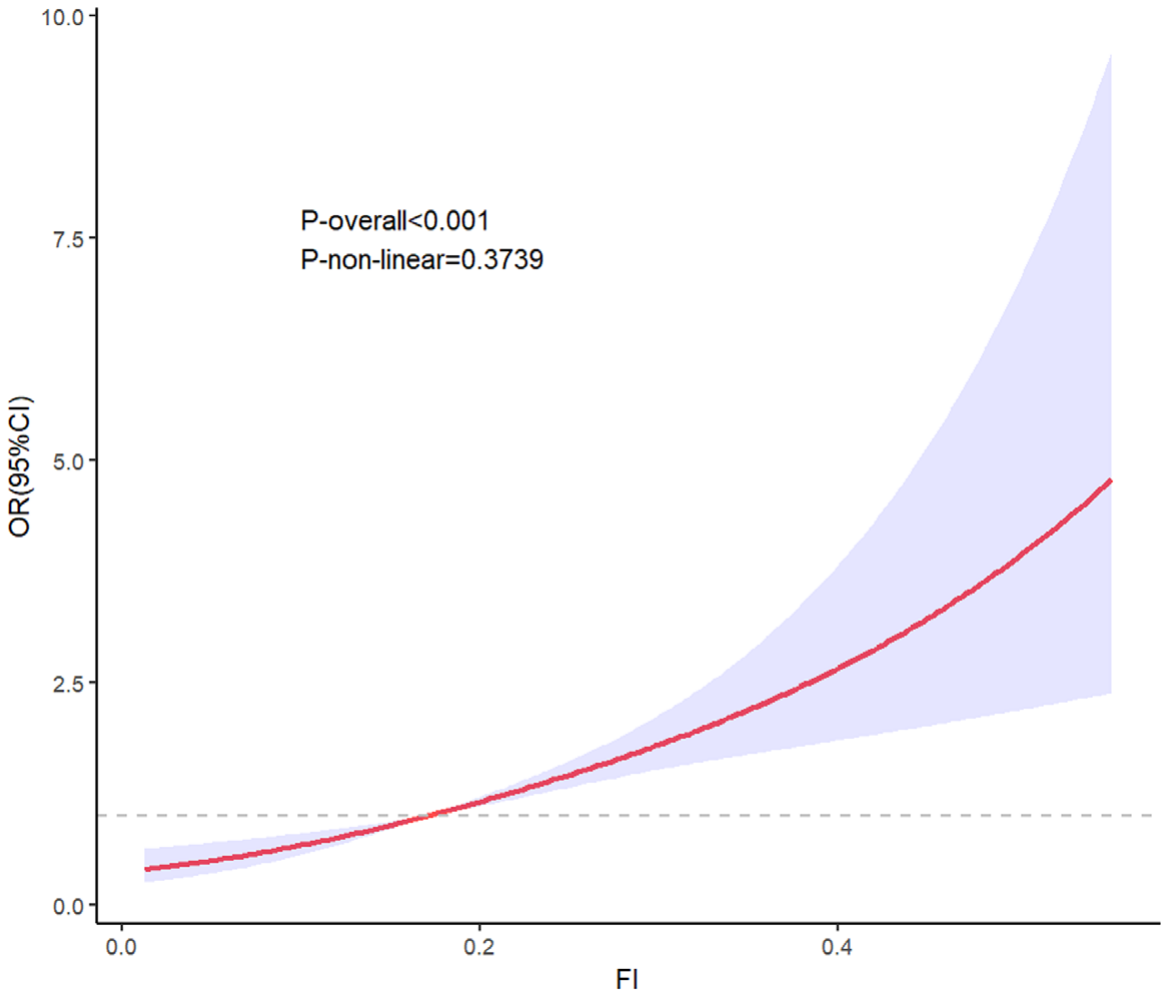
Restricted cubic spline fitting between FI and ED adjusted for age, race, marital status, education level, PIR, BMI, alcohol intaking, cigarette smoking, physical activity status, diabetes, hypertension, and cardiovascular disease. 95% CI = 95% confidence interval, BMI = body mass index, ED = erectile dysfunction, FI = frailty index, OR = odds ratio, PIR = poverty-income ratio.

Figure 2 illustrates the relationship between the FI and ED through curve fitting analysis using an RCS. The results show that *P*-overall < .001, indicating a significant overall association between FI and ED; *P* nonlinear = .3739, suggesting no obvious nonlinear relationship between the 2, with a closer approximation to a linear association. From the perspective of the graphical trend, as FI increases, the OR of ED shows an upward trend, and the CI gradually widens. This indicates that a higher FI is associated with a higher risk of ED, which is consistent with the conclusion of a “dose-response relationship” derived from previous subgroup analyses. It further verifies the linear association pattern that a more severe degree of frailty corresponds to a higher risk of ED, providing more intuitive evidence of the dose–response relationship for research on the association between FI and ED.

### 3.3. Subgroup analysis

**Figure 3.**
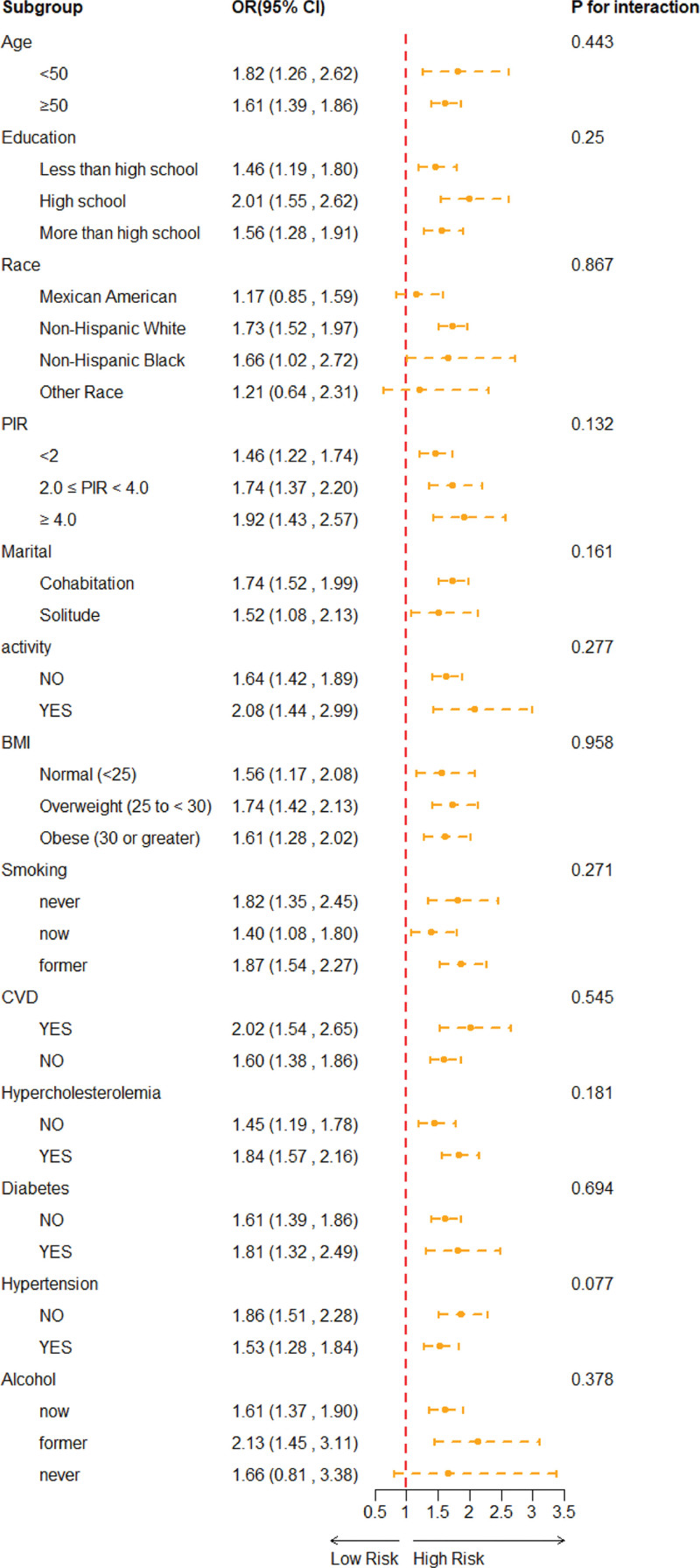
Subgroup analysis for FI and ED adjusted for age, race, marital status, education level, PIR, BMI, alcohol intaking, cigarette smoking, physical activity status, diabetes, hypertension, cardiovascular disease. Weighted. 95% CI = 95% confidence interval, BMI = body mass index, CVD = cardiovascular disease, ED = erectile dysfunction, FI = frailty index, OR = odds ratio, PIR = poverty-income ratio.

Figure 3 presents the stratified analysis and interaction effect test of the relationship between the FI and the risk of ED. The results show that in most subgroups, there was a significant positive association between FI and ED risk (OR > 1 and *P* < .05). Meanwhile, “*P* for interaction” was >.05 in all subgroups, indicating that the association between FI and ED risk was relatively consistent across different populations, meaning the relationship between FI and ED is robust.

### 3.4. Diagnostic performance of FI

**Figure 4.**
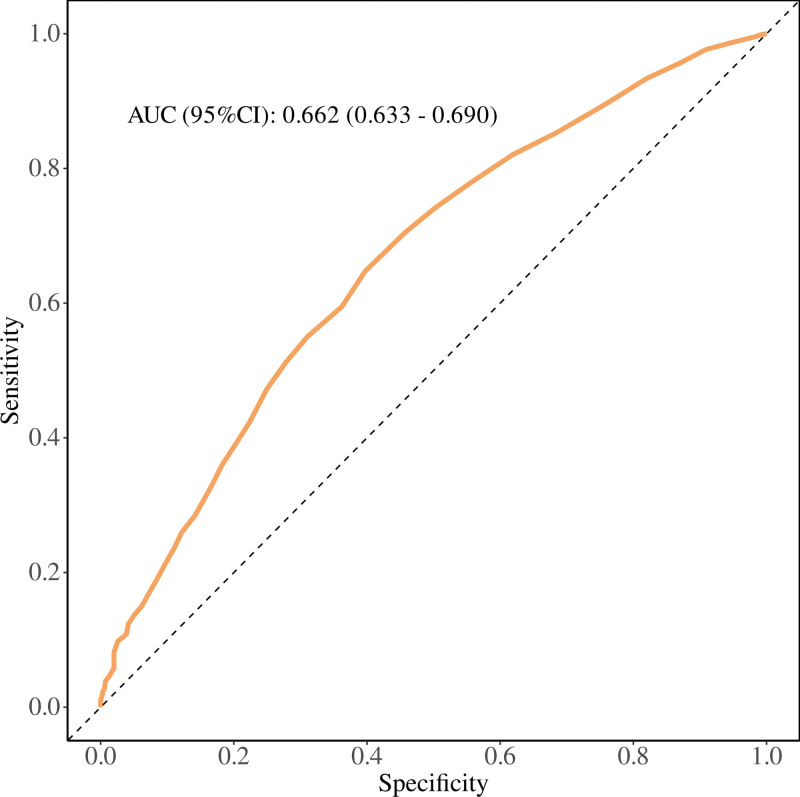
ROC curves and the AUC values of the FI in diagnosing ED. 95% CI = 95% confidence interval, AUC = area under the curve, ED = erectile dysfunction, FI = frailty index, ROC = receiver operating characteristic.

Figure 4 shows the ROC curve used to evaluate the predictive efficacy of the FI for ED. In this study, the area under the curve of the ROC curve for FI in predicting ED was 0.662 (95% CI: 0.633–0.690). This indicates that FI can identify the risk of ED to a certain extent, providing a reference for the prevention and early detection of ED.

## 4. Discussion

The results of this study indicate a significant positive correlation between the FI and ED, meaning that as the FI increases, the risk of ED shows an upward trend. This finding not only provides a new perspective for understanding the multidimensional pathogenic factors of ED but also offers important insights for the comprehensive management of men’s health.

In the context of existing research, the findings of this study are highly consistent with several previous explorations. Previous studies have demonstrated that frailty, as a syndrome characterized by decreased physiological reserve capacity across multiple systems, is closely associated with various age-related chronic diseases and dysfunctions. Meanwhile, ED, regarded as a “barometer” of male health, involves multiple mechanisms including vascular, neurological, endocrine, and psychological factors – these overlap considerably with the physiological systems affected by frailty.^[24,25]^ A study from China has shown a strong correlation between frailty and ED; however, our research, with a larger sample size and more detailed analytical methods, further elaborates on their association.^[26]^

A further analysis of the underlying mechanisms suggests that this positive association may be mediated through the following pathways. Chronic low-grade inflammation serves as a crucial common pathological basis: in a state of frailty, the body remains in a persistently activated inflammatory state, with elevated levels of inflammatory factors such as tumor necrosis factor-α and interleukin-6. These factors can impair vascular endothelial function and reduce the release of nitric oxide.^[27–29]^ Nitric oxide, however, is a key mediator of penile erection, and impairments in its synthesis and release directly affect cavernosal blood perfusion. Meanwhile, the enhanced oxidative stress often associated with frailty further exacerbates vascular endothelial damage, forming a vicious cycle of “inflammation → endothelial dysfunction → decreased erectile function.”^[30–32]^

Endocrine and metabolic disorders may also be involved: frail men often exhibit decreased testosterone levels. As a key hormone for maintaining penile erectile function, testosterone deficiency can lead to apoptosis of cavernous smooth muscle cells, impaired vasodilatory function, and simultaneously affect sexual desire and sexual drive.^[33–36]^ In addition to testosterone, other frailty-related endocrine changes (such as insulin resistance and abnormal growth hormone axis) may also play a role.^[37–39]^

A decline in multi-organ functional reserve constitutes another critical link: individuals with frailty exhibit reduced reserve capacity of the cardiovascular system and accelerated progression of atherosclerosis, which increases the risk of abnormal penile arterial hemodynamics.^[40,41]^ In addition, autonomic nervous system dysfunction may affect the neural regulation of penile erection, while physical function decline such as reduced muscle mass and decreased muscle strength may also indirectly impair the physical capacity to engage in sexual activity.^[42,43]^

From a clinical perspective, the findings of this study suggest that in the assessment and management of ED, attention should not be limited to the local genitourinary system; instead, the evaluation of overall health status should also be emphasized. As a simple and quantifiable tool for assessing overall health, the FI is expected to be used for screening high-risk groups for ED. For men with a higher degree of frailty, even those with mild ED symptoms, comprehensive interventions should be initiated as early as possible. These interventions include improving nutritional status, increasing resistance exercise to enhance muscle mass, and controlling chronic inflammation, with the aim of delaying or reversing the frailty process and thereby improving erectile function. In addition, for patients with refractory ED, assessing the frailty status may provide a basis for adjusting treatment plans. For example, while administering drug therapy, a multidisciplinary management approach targeting frailty can be combined.

Our study has several notable strengths. First, we used data from NHANES and applied sampling weights to enhance the representativeness of the sample. Second, we adjusted for a wide range of covariates, which strengthened the robustness of the results. Third, we conducted in-depth subgroup analyses based on different demographic and clinical characteristics, confirming the consistency of the findings across diverse populations. Finally, we visualized the dose–response relationship between the FI and ED risk using plots generated by the RCS.

However, our study also has certain limitations. First, the cross-sectional design cannot clarify the causal relationship between the 2. Whether frailty is a cause of ED or whether ED accelerates the progression of frailty still needs to be verified by longitudinal cohort studies. Second, the constituent indicators of the FI may vary among different study populations, and whether the indicators used in this study are applicable to other populations requires further verification. Third, the assessment of ED relies on self-reported data from survey participants, which may lead to an underestimation of the true prevalence of ED. Although we have adjusted for numerous potential confounding factors, we cannot rule out residual confounding from unmeasured or inaccurately measured variables. Fourth, the data utilized in this study were drawn from the period 2001 to 2004, which is now approximately 2 decades old, inevitably introducing a limitation regarding its temporal relevance. Therefore, the conclusions of this study must be interpreted with caution, and these limitations in interpreting the results should be acknowledged.

Future research can be carried out in 3 aspects: first, prospective cohort studies should be conducted to clarify the causal sequence between frailty and ED. Second, molecular biology studies are needed to explore in depth the common pathophysiological mechanisms of the 2, such as the specific roles of inflammatory factors and oxidative stress markers in this process. Third, randomized controlled trials should be designed to verify the improvement effect of frailty-targeted interventions (e.g., exercise interventions, nutritional supplements) on ED, so as to provide high-level evidence for clinical practice.

## 5. Conclusion

In conclusion, this study indicates a positive correlation between the FI and ED, and suggests that the FI has promising predictive efficacy for ED. It provides new clues for understanding the multidimensional etiologies of ED and opens up a new perspective for the comprehensive management of men’s health. Incorporating frailty assessment into the clinical practice of ED is expected to improve the accuracy and effectiveness of diagnosis and treatment, thereby enhancing patients’ quality of life.

## Acknowledgments

We would like to express our sincere gratitude to the Natural Science Foundation of Chongqing, China (Grant No. CSTB2023NSCQ-MSX0195), the Innovation Program for Chongqing’s Overseas Returnees (Grant No. cx2019146), and the Chongqing Health Appropriate Technology Promotion Project (Grant No. 2024jstg031) for their financial support, which made this study possible. We also thank all participants involved in the study and the research teams for their contributions.

## Author contributions

**Conceptualization:** Wei Wu, Peihe Liang.

**Data curation:** Wei Wu.

**Formal analysis:** Wei Wu.

**Investigation:** Wei Wu, Peihe Liang.

**Methodology:** Wei Wu, Peihe Liang.

**Project administration:** Wei Wu, Peihe Liang.

**Resources:** Wei Wu, Peihe Liang.

**Software:** Wei Wu.

**Validation:** Wei Wu.

**Visualization:** Wei Wu.

**Funding acquisition:** Peihe Liang.

**Writing – review & editing:** Peihe Liang.

**Writing – original draft:** Wei Wu.


