# Supplementary material for: Association between frailty index and erectile dysfunction: A cross-sectional study using NHANES 2001 to 2004 data
Source: Medicine (Baltimore). 2026 May 8;105(19):e48464. doi: 10.1097/MD.0000000000048464 (PMC13166479; doi:10.1097/MD.0000000000048464)
Supplement: Supplementary file 1 [file medi-105-e48464-s001.pdf]

Table S1. Variables in the 49-item frailty index and their respective scorings.

**Cognition**	
1.Experience confusion/memory problems	Yes=1, No=0
**Dependence**	
2.Managing money	Difficulty=1, No Difficulty=0
3.Stooping, crouching, kneeling	Difficulty=1, No Difficulty=0
4.Lifting or carrying	Difficulty=1, No Difficulty=0
5.House chore	Difficulty=1, No Difficulty=0
6.Preparing meals	Difficulty=1, No Difficulty=0
7.Standing up from armless chair	Difficulty=1, No Difficulty=0
8.Getting in and out of bed difficulty	Difficulty=1, No Difficulty=0
9.Using fork, knife, drinking from cup	Difficulty=1, No Difficulty=0
10.Dressing yourself	Difficulty=1, No Difficulty=0
11.Standing for long periods difficulty	Difficulty=1, No Difficulty=0
12.Grasp/holding small objects	Difficulty=1, No Difficulty=0
13.Attending social event	Difficulty=1, No Difficulty=0
14.Push or pull large objects	Difficulty=1, No Difficulty=0
15.walking for a quarter mile difficulty	Difficulty=1, No Difficulty=0
16.walking up 10 steps difficulty	Difficulty=1, No Difficulty=0
**Depressive Symptoms**	
17.Have little interest in doing things	Nearly every day=1, More than half the days=0.66, Several days=0.33, Not at all=0
18.Feeling down, depressed, or hopeless	Nearly every day=1, More than half the days=0.66, Several days=0.33, Not at all=0
19.Trouble sleeping or sleeping too much	Nearly every day=1, More than half the days=0.66, Several days=0.33, Not at all=0
20.Feeling tired or having little energy	Nearly every day=1, More than half the days=0.66, Several days=0.33, Not at all=0
21.Poor appetite or overeating	Nearly every day=1, More than half the days=0.66, Several days=0.33, Not at all=0
22.Feeling bad about yourself	Nearly every day=1, More than half the days=0.66, Several days=0.33, Not at all=0
23.Trouble concentrating on things	Nearly every day=1, More than half the days=0.66, Several days=0.33, Not at all=0
**Comorbidities**	
24.Arthritis	Yes=1, Suspect=0.5, No=0
25.Thyroid problems	Yes=1, Suspect=0.5, No=0
26.Chronic bronchitis	Yes=1, Suspect=0.5, No=0
27.Cancer	Yes=1, Suspect=0.5, No=0
28.Congestive heart failure	Yes=1, Suspect=0.5, No=0
29.Coronary heart disease	Yes=1, Suspect=0.5, No=0
30.Angina	Yes=1, Suspect=0.5, No=0
31.Heart attack	Yes=1, Suspect=0.5, No=0
32.Stroke	Yes=1, Suspect=0.5, No=0

33.Blood pressure	Yes=1, Suspect=0.5, No=0	
34.Diabetes	Yes=1, Suspect=0.5, No=0	
35.Weak/failing kidneys	Yes=1, Suspect=0.5, No=0	
36.Urinary leakage	Yes=1, Suspect=0.5, No=0	
**Hospital Utilization and Access to Care**		
37.Self-rated health	Fair, poor=1, Excellent, Very good, good=0	
38.Health now compared 1 year ago	Worse=1, About the same, Better=0	
39.Overnight hospital patient in past year	Yes=1, No=0	
40.Frequency of healthcare using during past year	None=0, 1-5=0.5, 5 and more than 5=1	
41.Number of prescribed medications	None=0, 1-4=0.5, More than 5=1	
**Physical Performance and Anthropometry**		
42.Body mass index	<18.5, ≥30=1	
	25-<30=0.5	
	18.5-25=0	
43.Handgrip Strength	MALE:	FEMALE:
	For BMI⩽24, GS⩽29	For BMI⩽23, GS⩽17
	For BMI 24.1-28, GS⩽30	For BMI 23.1-26, GS⩽17.3
	For BMI＞28, GS⩽32=1	For BMI26.1-29, GS⩽18
**Laboratory Values**		For BMI＞29, GS⩽21=1
44. Glycohemoglobin (%)	0%-5.7%=0,>5.7%=1	
45.Red blood cell count (million cells/μL)	M: 4.7-6.1=0, Other=1; F: 4.2-5.4=0, Other=1	
46.Hemoglobin (g/dL)	M:13.5-18=0, Other=1; F: 12-16=0, Other=1	
47.Red cell distribution width (%)	11.6-14.6=0, Other=1	
48.Lymphocyte percent(%)	20-40=0, Other=1	
49.Segmented neutrophils percent(%)	40-80=0, Other=1	

BMI, Body mass index; GS, grip strength.
